# Targeted TLR9-activation/STAT3-blocking abrogates immunosuppressive functions of myeloid-derived suppressor cells from late-stage prostate cancer patients

**DOI:** 10.1186/2051-1426-2-S3-P104

**Published:** 2014-11-06

**Authors:** Dewan Md Sakib Hossain, Dayson Moreira, Qifang Zhang, Stephen J Forman, Sumanta Pal, Marcin Kortylewski

**Affiliations:** 1 City of Hope, Duarte, Duarte, CA, USA

## 

Recent clinical advances underscored that elimination of tumor-induced immunosuppression is prerequisite for successful therapy of advanced human cancers. The myeloid-derived suppressor cells (MDSCs) are one of the major populations limiting T cell-mediated antitumor immune responses in late-stage solid tumors, such as castration-resistant prostate cancers (CRPCs). Targeting MDSCs proved challenging due to their heterogeneity and phenotypic similarities with other myeloid cell lineages. Here, we identify a population of granulocytic MDSCs, which accumulate in patients' blood during progression of prostate cancer from localized to metastatic disease. The CRPC-associated MDSCs potently inhibit proliferation, IFN-γ and Granzyme-B production in autologous CD8^+ ^T cells. Importantly, the MDSCs in blood and in lymph nodes of CRPC patients show high levels of activated STAT3 transcription factor, which is a crucial immune checkpoint regulator. We previously generated an original method to silence genes specifically in TLR9^+ ^myeloid cells using CpG-siRNA conjugates [1,2]. Now, we demonstrate that the CpG-*STAT3*siRNA is also effectively internalized by human MDSCs and induces *STAT3 *silencing without the need for transfection reagents. Our further studies demonstrated that two-pronged TLR9-activation/STAT3-blocking by CpG-*STAT3*siRNA inhibits immunosuppressive effects of CRPC-MDSCs on CD8^+ ^T cells, while both TLR9 triggering or STAT3 inhibition alone have only minimal effects. The reversal of MDSC immunosuppression by CpG-*STAT3 *siRNA results from reduced expression and enzymatic activity of Arginase I, a downstream STAT3 target gene. Overall, our study demonstrates the feasibility of overcoming immunosuppression in CRPC patients using CpG-*STAT3*siRNA strategy to alleviate effects of MDSCs. We anticipate that our strategy to reverse MDSC-mediated immunosuppression without depleting myeloid cell populations will generate safer and more effective immunotherapy for late-stage, metastatic prostate cancers and potentially other solid tumors.

*This project described was supported by the National Cancer Institute of the National Institutes of Health under award number R01CA155367 and by the Department of Defense Prostate Cancer Research Program under award number W81XWH-12-1-0132 to M.K*.
